# Altered L-Arginine Metabolic Pathways in Gastric Cancer: Potential Therapeutic Targets and Biomarkers

**DOI:** 10.3390/biom11081086

**Published:** 2021-07-23

**Authors:** Iwona Bednarz-Misa, Mariusz G. Fleszar, Paulina Fortuna, Łukasz Lewandowski, Magdalena Mierzchała-Pasierb, Dorota Diakowska, Małgorzata Krzystek-Korpacka

**Affiliations:** 1 Department of Biochemistry and Immunochemistry, Wroclaw Medical University, 50-368 Wroclaw, Poland; iwona.bednarz-misa@umed.wroc.pl (I.B.-M.); fleszar.mariusz@gmail.com (M.G.F.); paulina.fortuna@umed.wroc.pl (P.F.); lukasz.lewandowski@umed.wroc.pl (Ł.L.); magdalena.mierzchala-pasierb@umed.wroc.pl (M.M.-P.); 2 Department of Gastrointestinal and General Surgery, Wroclaw Medical University, 50-368 Wroclaw, Poland; dorota.diakowska@umed.wroc.pl; 3 Department of Nervous System Diseases, Wroclaw Medical University, 51-618 Wroclaw, Poland

**Keywords:** metabolic reprogramming, arginine auxotrophy, argininosuccinate lyase, dimethylarginine dimethylaminohydrolase, protein arginine methyltransferase, nitric oxide synthase, dimethylarginine, ornithine decarboxylase, ornithine translocase, argininosuccinate synthase

## Abstract

There is a pressing need for molecular targets and biomarkers in gastric cancer (GC). We aimed at identifying aberrations in L-arginine metabolism with therapeutic and diagnostic potential. Systemic metabolites were quantified using mass spectrometry in 293 individuals and enzymes’ gene expression was quantified in 29 paired tumor-normal samples using qPCR and referred to cancer pathology and molecular landscape. Patients with cancer or benign disorders had reduced systemic arginine, citrulline, and ornithine and elevated symmetric dimethylarginine and dimethylamine. Citrulline and ornithine depletion was accentuated in metastasizing cancers. Metabolite diagnostic panel had 91% accuracy in detecting cancer and 70% accuracy in differentiating cancer from benign disorders. Gastric tumors had upregulated *NOS2* and downregulated *ASL*, *PRMT2*, *ORNT1*, and *DDAH1* expression. *NOS2* upregulation was less and *ASL* downregulation was more pronounced in metastatic cancers. Tumor *ASL* and *PRMT2* expression was inversely related to local advancement. Enzyme up- or downregulation was greater or significant solely in cardia subtype. Metabolic reprogramming in GC includes aberrant L-arginine metabolism, reflecting GC subtype and pathology, and is manifested by altered interplay of its intermediates and enzymes. Exploiting L-arginine metabolic pathways for diagnostic and therapeutic purposes is warranted. Functional studies on *ASL*, *PRMT2*, and *ORNT1* in GC are needed.

## 1. Introduction

Gastric cancer remains the leading cause of cancer-related deaths despite a steady decline in incidence rates, attributed to improved hygiene and diet and the popularization of therapies against *Helicobacter pylori* [1]. While efficient in reducing the occurrence of non-cardia subtype of gastric adenocarcinoma (GA), they failed to stop the rising incidence of cardia subtype (CA) [2]. Poor survival rates characteristic for gastric cancer result from the disease being diagnosed at advanced stage, when it is not amenable for curative resection, leaving chemotherapy as a major therapeutic option [1]. However, chemotherapy is not only highly toxic but its effectiveness is below expectations. Therefore, “a radical shift toward precision medicine” is advocated. However, this requires unraveling the molecular landscape of gastric cancer in order to establish novel therapeutic targets and biomarkers [2].

Reprogramming of metabolic pathways is a well-recognized hallmark of cancer [3], going well beyond the Warburg effect [4]. Therefore, untargeted and targeted metabolic profiling is viewed as a promising tool in precision medicine [5]. However, recent advances in metabolomics showed that the alterations are neither uniform across cancer types nor constant over time. Rather, they reflect cancer heterogeneity as well as its progression [6].

L-arginine (Arg) metabolic pathways are among those repeatedly found to be deregulated [7,8,9]. Pharmacological manipulation of pathway enzymes is viewed as an attractive therapeutic approach while monitoring enzyme activity and/or metabolite concentration may aid cancer diagnosis and treatment [10,11,12,13]. However, a better understanding of pathway status and function in cancer is needed as even the role of arginine, the pathway precursor, is ambiguous [13]. Arginine is competed for by immune and cancer cells, either contributing to immunosurveillance or supporting tumor growth and metastasis [13]. Still, the amino acid role in gastric cancer might not be unequivocally tumor-supporting. Uncharacteristically, arginine has been shown to inhibit growth of gastric cancer cells in vitro by upregulating caspase 8 expression and consequently inducing apoptosis [14].

**Figure 1:**
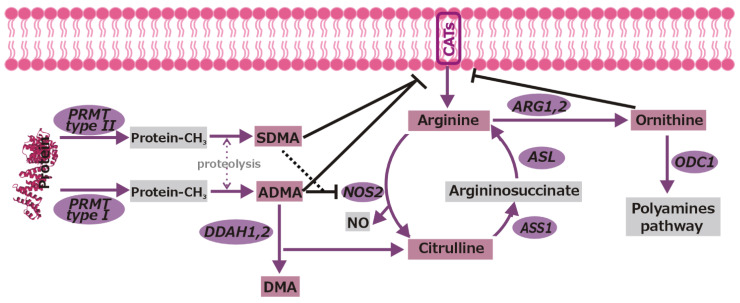
Overview of metabolic pathways of L-arginine. Pathway players not investigated in the current study are indicated by the grey color, while the analyzed pathway metabolites are depicted in rectangular frames, and enzymes in elliptical frames. Inhibitory effects are marked by black blunt-ended arrows—dashed if the effect is weak. ADMA, asymmetric dimethylarginine; SDMA, symmetric dimethylarginine; DMA, dimethylamine; ASL, argininosuccinate lyase; ARG1,2, arginase 1 and 2; ASS1, argininosuccinate synthase 1; CATs, cationic amino acid transporters; DDAH1,2, dimethylarginine dimethylaminohydrolase 1 and 2; NO, nitric oxide; NOS2, nitric oxide synthase 2; ODC1, ornithine decarboxylase 1; PRMT, protein arginine methyltransferase.

Synthesis of NO by NO synthases (NOSs) and synthesis of L-ornithine (ornithine; Orn) by arginases (ARGs), as a precursor of polyamines synthesized by ornithine decarboxylase (ODC), are two main competitive ways of Arg utilization [11]. NO synthesis is inhibited by methylated arginine derivatives such as asymmetric and symmetric dimethylarginines (ADMA and SDMA), products of protein arginine methyltransferases (PRMTs), as they compete with Arg for NOS as well as for amino acid transporters. The ADMA is metabolized to L-citrulline (citrulline; Cit) and dimethylamine (DMA) by dimethylarginine dimethylaminohydrolases (DDAHs). Citrulline can be recycled to arginine in a two-step reaction catalyzed by argininosuccinate synthase-1 (ASS1) and argininosuccinate lyase (ASL) [10,15,16]. A basic overview of key players of arginine metabolic pathways and their interrelationship is depicted in Figure 1.

Considering the pressing need for new molecular targets and biomarkers for gastric cancer and the growing interest in Arg metabolism in this capacity, our aim was to explore the pathway status in gastric cancer in order to identify aberrations with therapeutic and diagnostic potential. In the present study, a comprehensive analysis of systemic metabolite concentration (Arg, Cit, Orn, ADMA, SDMA, and DMA) and local enzyme (*ASL*, *ARG1*, *ARG2*, *ASS1*, *DDAH1*, *DDAH2*, *NOS2*, *ODC1*, *PRMT1*, *PRMT2*, and *PRMT5*) and transporter (*ORNT1*) expression was conducted. Metabolomic and transcriptomic data were referred to cancer anatomical subsite and pathology. Patterns of interrelationships of pathway players and their correlation with local and systemic immune, inflammatory, and angiogenic mediators and other molecules relevant for cancer were examined.

## 2. Materials and Methods

### 2.1. Patients and Controls

#### 2.1.1. Metabolomic Analysis

Biobanked serum samples stored at −80 °C, obtained from 293 individuals, including 153 apparently healthy controls, 50 patients with benign gastric disorders, and 90 patients with histopathologically confirmed gastric adenocarcinoma, were used in metabolomic analysis. Cancer patients and patients with benign gastric disorders (gastritis, cardiospasmus, gastro-esophageal reflux disease) were admitted to the Department of Gastrointestinal and General Surgery of Wroclaw Medical University for the disease diagnosis and/or treatment. Cancer patients underwent standard preoperative evaluation consisting of blood work, physical examination, and imaging techniques (ultrasonography, computed tomography, and magnetic resonance). Cancers were staged clinically using the 7th edition of the Union for International Cancer Control TNM system. Control individuals were recruited from apparently healthy blood donors. Detailed population characteristics are summarized in Table 1.

#### 2.1.2. Transcriptomic Analysis

Transcriptomic analysis was conducted using biobanked material—tissue fragments from tumor and patient-matched macroscopically normal tumor-adjacent mucosa soaked in RNAlater (Ambion Inc., Austin TX, USA) and stored in −80 °C—obtained from 29 patients with histopathologically confirmed gastric adenocarcinoma, submitted to the Department of Gastrointestinal and General Surgery of Wroclaw Medical University for curative resection. Patients from whom samples were collected did not have any severe systemic illness or gross metastatic disease and were not subjected to prior radio- or chemotherapy. Patients underwent standard preoperative evaluation consisting of blood work, physical examination, and imaging techniques (ultrasonography, computed tomography, and magnetic resonance). Cancers were rated pathologically using the 7th edition of the Union for International Cancer Control TNM system. In all cases, the resection margins have been confirmed to be tumor-free. Detailed population characteristics are summarized in Table 2.

### 2.2. Analytical Methods

#### 2.2.1. Metabolomic Analysis

##### Chemicals and Reagents

LC/MS grade acetonitrile, methanol, and water were obtained from Merck Millipore (Warsaw, Poland). Analytical standards: hydrochloride salts of unlabeled dimethylamine (D0-DMA), hexadeutero- dimethylamine (D6-DMA, declared as 99 atom % 2H), L-arginine, SDMA, ADMA, L-citrulline, L-ornithine monohydrochloride, labeled L-ornithine hydrochloride (3,3,4,4,5,5-D6-ornithine), benzoyl chloride (BCl) and sodium tetraborate were procured from Sigma-Aldrich (Poznan, Poland). Isotope labeled L-arginine:HCl (D7-arginine, 98%) and asymmetric dimethylarginine (2,3,3,4,4,5,5-D7-ADMA, 98%) were obtained from Cambridge Isotope Laboratories (Tewksbury, MA, USA). Leucine–enkephalin was purchased from Waters (Milford, MA, USA).

##### Sample Extraction

The extraction and derivatization of metabolites associated with Arg metabolic pathways were carried out using the previously described method [17]. One hundred microliters of calibration standards or serum samples were mixed with 50 µL of borate buffer (pH = 9.2) and 10 µL of internal standard solution of D6-arginine, D6-DMA, D7-ADMA, D6-ornithine (100 μM, 50 μM, 20 μM, 100 μM, and 70 μM, respectively). After mixing, 400 µL of acetonitrile and 10 µL of 10% BCl in acetonitrile were added. Then, the mixture was vortexed for 10 min at 25 °C. The samples were centrifuged at 10,000 rpm for 7 min at 4 °C. Obtained supernatants were diluted 4:1 with water.

##### LC-QTOF-MS Analysis

The LC-QTOF-MS system consisted of Acquity UPLC system (Waters, Milford, MA, USA) and quadrupole time-of-flight mass spectrometer (Xevo G2 Q-TOF MS, Waters, Milford, MA, USA).

Chromatographic conditions used for compound quantification were as follow: Waters HSS T3 chromatographic column (1.8 µm, 1.0 × 50 mm) with mobile phase A = 0.1% formic acid in water and mobile phase B = 0.1% formic acid in methanol, column temperature set at 60 °C, flow rate = 0.220 mL/min and injection volume of 2 µL. Typical elution conditions were held at 5% B from 0.0 to 1 min, to 14% B in 2.5 min, to 60% B in 1.5 min, to 90% B in 0.5 min, held at 90% B for 1.1 min, to 5%B in 0.1 min, held at 5% B for 1.9 min.

MS acquisition was carried out with an electro spray ionization (ESI) ion source operated in a positive mode. Source parameters were as follows: nebulizing and drying gas (nitrogen): 650 L/h and 65 L/h, respectively; spray voltage: 0.5 kV; source temperature: 120 °C, and the desolvation temperature: 400 °C. The scan range was 150–650 *m/z* for all acquisition events. The target metabolites were quantified based on their extracted ion chromatograms and *m/z* for each compound were as follows: ornithine: 237.1239; D6-ornithine 243.1339; arginine: 279.1457; D7-arginine: 286.1897; ADMA and SDMA: 307.1770; D7-ADMA: 314.2209; citrulline: 263.1090; D4-cytrulline: 267.1382; DMA: 150.0919 and D6-DMA: 156.1295.

#### 2.2.2. Transcriptomic Analysis

RNA was isolated from 30–40 mg tissue fragments homogenized in lysis buffer (part of PureLink™ RNA Mini Kit from Thermo-Fisher Scientific, Waltham, MA, USA) with addition of β-mercaptoethanol (Sigma-Aldrich, St. Luis, MO, USA) in Fastprep 24 Homogenizer (MP Biomedical, Solon, OH, USA) using ceramic spheres.

Phenol-chloroform extraction was used for RNA isolation and RNA isolates were additionally purified with PureLink™ RNA Mini Kit (Thermo-Fisher Scientific) and subjected to on-column genomic DNA digestion with DNase PureLink™ DNase Set (PureLink™ DNase Set, Thermo-Fisher Scientific). RNA concentration, purity and integrity were determined spectrophotometrically using Nanodrop 2000 (Thermo-Fisher Scientific) and by microfluidic electrophoresis using the Experion platform and dedicated RNA StdSens analysis kits (BioRad, Hercules, CA, USA).

The cDNA library was created from 1000 ng of RNA, reversely transcribed in C1000 thermocycler (BioRad) using iScript™ cDNA Synthesis Kit (BioRad) according to the manufacturer’s recommendations.

The qPCRs were conducted on diluted cDNA (1:5) using 1 µL of each 10 nM forward and reverse target-specific primers and SsoFast EvaGreen^®^ Supermix (BioRad) in CFX96 Real-Time PCR system (BioRad). The cycling conditions were as follows: 30 s activation at 95 °C, 5 s denaturation at 95 °C, annealing/extension for 5 s at 61 °C, 40 cycles, followed by melting step (60–95 °C with fluorescent reading every 0.5 °C). Primers were synthesized by Genomed (Warsaw, Poland), based on sequences proposed by OriGene (Rockville, MD, USA), and their specificity was tested by melting curve analysis and using an electrophoresis in a high-resolution agarose (SeaKem LE agarose from Lonza, Basel, Switzerland) in TBE with SYBR Green (Lonza) detection. The following primer sequences were used: 5′-ctctcaacagcatggatgccac-3′ (forward) and 5′-cttggtgcagtagaggatgagg-3′ (reverse) for *ASL* (amplicon size: 122 base pairs (bp)); 5′-tcatctgggtggatgctcacac-3′ (forward) and 5′-gagaatcctggcacatcgggaa-3′ (reverse) for *ARG1* (amplicon size: 130 bp); 5′-ctggcttgatgaaaaggctctcc-3′ (forward) and 5′-tgagcgtggattcactatcaggt-3′ (reverse) for *ARG2* (amplicon size: 119 bp); 5′-gctgaaggaacaaggctatgacg-3′ (forward) and 5′-gccagatgaactcctccacaaac-3′ (reverse) for *ASS1* (amplicon size: 155 bp); 5′-gctctacacctccaatgtgacc-3′ (forward) and 5′-ctgccgagatttgagcctcatg-3′ (reverse) for *NOS2* (amplicon size: 136 bp); 5′-ccaaagcagtctgtcgtctcag-3′ (forward) and 5′-cagagattgcctgcacgaaggt-3′ (reverse) for *ODC* (amplicon size: 162 bp); 5′-ggagacatcagggaagatagcc-3′ (forward) and 5′-gctcagttcatagccaccgaag-3′ (reverse) for *ORNT1* (amplicon size: 163 bp); 5′-atgcagtctccacagtgccagt-3′ (forward) and 5′-ttgtcgtagcggtggtcactca-3′ (reverse) for *DDAH1* (amplicon size: 151 bp); 5′-ctttcttcgtcctgggttgcct-3′ (forward) and 5′-ctccagttctgagcaggacaca-3′ (reverse) for *DDAH2* (amplicon size: 136 bp); 5′-tgcggtgaagatcgtcaaagcc-3′ (forward) and 5′-ggactcgtagaagaggcagtag-3′ (reverse) for *PRMT1* (amplicon size: 142 bp); 5′-gcagttggacatgagaaccgtg-3′ (forward) and 5′-aggctctggaagtggacgctaa-3′ (reverse) for *PRMT2* (amplicon size: 129 bp); 5′-ctagaccgagtaccagaagagg-3′ (forward) and 5′-cagcatacagctttatccgccg-3′ (reverse) for *PRMT5* (amplicon size: 136 bp). Primers for *GAPDH*, used as normalizer, were designed using Beacon Designer Probe/Primer Design Software (BioRad), validated in silico by Blast analysis: 5′-tagattattctctgatttggtcgtattgg-3′ (forward) and 5′-gctcctggaagatggtgatgg-3′ (reverse); amplicon size of 223 bp.

Prior to the statistical analysis, technical qPCR replicates were averaged. Geometric mean of all Cq values for each gene was calculated. Individual sample Cq were subtracted from mean giving ΔCq, linearized by 2^^ΔCq^ conversion, and subsequently normalized to *GAPDH* giving normalized relative quantities (NRQ) [18].

For the purpose of correlation analysis, previously published [19] data regarding tumor expression of representative cancer-related genes (*Ki67*, *HIF1A*, *BCLXL*, *CDKN1A*, *CCL2*, *PTGS2*, *GLUT1*, *VEGFA*, *CLDN2*, and *TJP1*) were retrieved from patients’ database.

#### 2.2.3. Immunoassays

For the purpose of correlation analysis, data concerning serum concentration of 48 cytokines and growth factors, measured using Luminex xMAP^®^ technology on the BioPlex 200 platform (Bio-Rad, Herkules CA, USA) and Panel I (27-plex) and Panel II (21-plex) Bio-Plex Pro™ Human Cytokine, Chemokine, and Growth Factor Magnetic Bead–Based Assays, were retrieved from patient’s database [19]. The following cytokines were quantified: eotaxin, IL-1β, IL-1ra, IL-2, IL-4, IL-5, IL-6, IL-7, IL-8, IL-9, IL-10, IL-12p70, IL-13, IL-15, IL-17, IFNγ, IP-10, FGF-2, G-CSF, GM-CSF, MCP-1, MIP-1α, MIP-1β, PDGF-BB, RANTES, TNFα, and VEGF-A as a 27-plex and IL-1α, IL-2Rα, IL-3, IL-12p40, IL-16, IL-18, CTACK, GRO-α, HGF, IFN-α2, LIF, MCP-3, M-CSF, MIF, MIG, β-NGF, SCF, SCGF-β, SDF-1α, TNF-β, and TRAIL as a 21-plex. All analyses were conducted in duplicate and following assay protocols. Data were analyzed using BioPlex Manager 6.0 software based on standard curves drawn using 5-PL logistic regression. Cytokines from 27-plex were determined in 82 patients and those from 21-plex in 26 patients. Some cytokines yielded values below the assays’ limit of detection; therefore, the exact number of analyzed cases was given along with results of correlation analysis.

### 2.3. Statistical Analysis

Data were assessed for normality and homogeneity of variances prior to each analysis using the Kolmogorov–Smirnov and Levene tests, respectively, and log-transformation was applied if appropriate. Expression data were examined using t-test for paired samples or Wilcoxon test (paired analysis) and with t-test for independent samples or Mann–Whitney U test and presented as, respectively, geometric means or medians with 95% confidence intervals (*CI*). In metabolomic analysis, two-group comparisons were conducted using t-test for independent samples with Welch correction in case of unequal variances, while multi-group comparisons using one-way analysis of variance with Scheffe post-hoc test or Kruskal-Wallis H test with Conover post-hoc test. Data were presented as means or geometric means with 95%*CI* or standard deviation or medians with 95%*CI*. Correlation analysis was conducted using Spearman rank correlation or Pearson correlation. The following descriptors were used for interpretation of correlation coefficients: <0.1 as negligible correlation; 0.1–0.39 as weak; 0.4–0.69 as moderate; 0.7–0.89 as strong; 0.9–1.0 as very strong correlation (as quoted in [20]). The ROC curve analysis was conducted to determine the strength of association and the diagnostic potential of assessed analyte. The ROC analysis data are reported as area under ROC curve (AUC), representing marker accuracy expressed in %. In addition, marker sensitivity and specificity were calculated. Enter method of logistic regression was applied to calculate predicted probabilities, subsequently used as dependent variable in ROC analysis of diagnostic potential of metabolite panels. Frequency analysis was conducted using Fisher’s exact test or Chi-square test. Multivariate analysis (stepwise method) was used to select independent predictors of gene expression.

All calculated probabilities were two-tailed. The *p* values ≤ 0.05 were considered statistically significant. The entire analysis was conducted using MedCalc^®^ Statistical Software version 19.6 (MedCalc Software Ltd., Ostend, Belgium; https://www.medcalc.org; 2020).

## 3. Results

### 3.1. Serum Concentrations of Arg/NO Pathway Metabolites

#### 3.1.1. Pathway Status in Gastric Cancers and Benign Disorders

**Figure 2:**
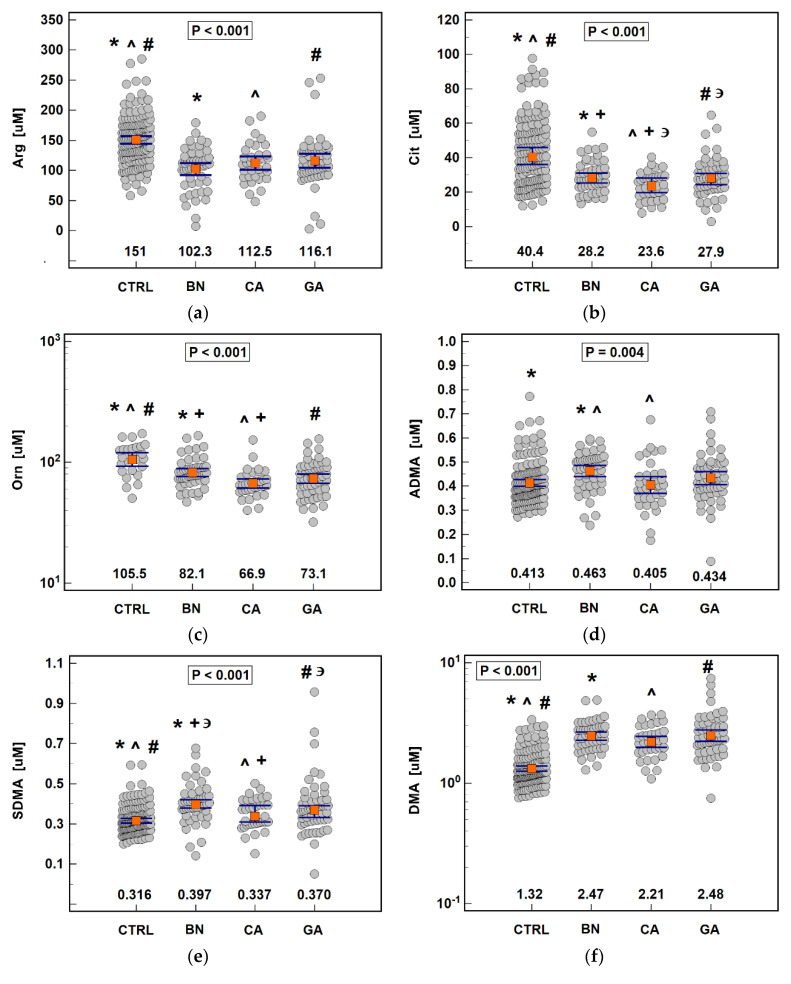
Serum concentrations of metabolites associated with arginine metabolism in patients with gastric cancers or benign disorders: (**a**) Arginine (Arg); (**b**) Citrulline (Cit); (**c**) Ornithine (Orn); (**d**) Asymmetric dimethylarginine (ADMA); (**e**) Symmetric dimethylarginine (SDMA); (**f**) Dimethylamine (DMA). Data presented as dot-plots with means (Arg and ADMA), geometric means (Orn and DMA) or medians (Cit and SDMA), accompanied by 95% confidence interval (orange squares with whiskers). Data were analyzed using one-way analysis of variance with Scheffe post-hoc test (Arg, Orn, ADMA, and DMA) or Kruskal–Wallis H test with Conover post-hoc test (Cit and DMA). Groups differing significantly in a post-hoc analysis (*p* < 0.05) are indicated by the same type of symbol: *, #, ^, +, or **∍**. CTRL, controls; BN, benign gastric disorders; CA, cardia subtype of gastric adenocarcinoma; GC, non-cardia subtype of gastric adenocarcinoma.

The concentration of all metabolites differed significantly between groups (Figure 2). Arginine, citrulline, and ornithine were significantly higher and SDMA and DMA were significantly lower in controls than in patients with cancer or benign gastric disorders, while ADMA was significantly higher, only compared to patients with benign conditions. Cancer patients had lower SDMA than those with benign disorders and those with CA also had lower citrulline, ornithine, and ADMA. Cancer patients with tumor location in gastric cardia had lower citrulline than those with non-cardia gastric tumors.

#### 3.1.2. Association with Cancer Pathology

Arginine, SDMA, and DMA were not associated significantly with cancer pathology. Ornithine and ADMA were weakly negatively correlated with overall TNM stage and with depth of tumor invasion. Citrulline was significantly lower in patients with lymph node and distant metastases and ornithine was also lower in the case of lymph node involvement (Table 3).

#### 3.1.3. Interplay between Pathway Metabolites, Cytokines and Growth Factors

The inter-relationship between pathway metabolites changes depending on health status. In controls, the strongest relationships were between ADMA and SDMA (r = 0.61, *p* < 0.0001), ornithine and citrulline (r = 0.60, *p* < 0.001), SDMA and DMA (r = 0.61, *p* < 0.0001), and ADMA and DMA (r = 0.58, *p* < 0.0001), all positive and moderate. Those associations weakened or disappeared in patients with benign gastric disorders (r = 0.39, *p* < 0.01 for ADMA and SDMA and r = 0.43, *p* < 0.01 for ornithine and citrulline). In turn, a weak positive correlation between ADMA and ornithine (r = 0.36, *p* < 0.01) and a moderate negative correlation between arginine and ornithine (r = −0.40, *p* < 0.01) occurred. In cancer patients, the correlation pattern was remodeled: SDMA and DMA were strongly positively correlated (r = 0.72, *p* < 0.0001) and moderate positive correlation occurred between citrulline and ADMA (r = 0.49, *p* < 0.0001), SDMA (r = 0.48, *p* < 0.0001), and DMA (r = 0.47, *p* < 0.0001). Weak positive associations were observed between DMA and ADMA (r = 0.30, *p* < 0.01), citrulline and ornithine (r = 0.31, *p* < 0.01), arginine and ADMA (r = 0.28, *p* < 0.01), and ornithine and ADMA (r = 0.27, *p* < 0.01).

The correlation patterns between metabolites associated with arginine metabolism and mediators of inflammatory and immune responses as well as angiogenesis were examined. Correlations found statistically significant are summarized in Table 4. Arginine was moderately negatively correlated with IL-2, IL-15, IL-16, MIF, and SDF1α and citrulline with IL-18 and TRAIL. Ornithine displayed moderate positive correlation with IL-16 and negative with IL-17. ADMA and SDMA were moderately positively correlated with HGF and SDMA also with SCF. DMA was moderately negatively related to MIF and positively to SCGFβ. The remining correlations were weak (ρ < 0.4).

#### 3.1.4. Diagnostic Significance of the Pathway

Receiver operating characteristic (ROC) curve analysis was employed to determine the strength of detected associations and diagnostic potential of metabolites associated with arginine metabolism, individually and as a panel. As a discriminator between healthy individuals and cancer patients, DMA and ornithine had high (over 80%) accuracy and their sensitivity was superior over specificity. The “all metabolites” panel had 91% accuracy and was characterized by high sensitivity and specificity. The panel also had superior 70% accuracy, accompanied by fair specificity and sensitivity, in discriminating cancer patients from those with benign gastric disorders. Citrulline was the only metabolite with statistically significant power in discriminating CA and GA patients and the panel had only slightly better accuracy (66% compared to 63% of citrulline) but had improved specificity (Table 5).

### 3.2. Local Expression of Enzymes Associated with Arginine Metabolism

#### 3.2.1. Pathway Enzymes in Gastric Cancers

To discern whether altered status of Arg metabolic pathways at systemic level is underlined by local alterations in expression patterns of genes encoding key pathway enzymes, the expression of *ASL*, *ARG1*, *ARG2*, *ASS1*, *DDAH1*, *DDAH2*, *NOS2*, *ODC1*, *ORNT1*, *PRMT1*, *PRMT2*, and *PRMT5* was examined.

Compared to unaltered mucosa, *NOS2* was upregulated in tumors by 16.4-fold on average, while *ASL* (by 1.9-fold), *DDAH1* (by 1.6-fold), *ORNT1* (by 2.5-fold), and *PRMT2* (by 1.7-fold) were downregulated (Table 6).

Using a two-fold change in expression as an arbitrary threshold for up- or downregulation, 52% of patients downregulated and 17% upregulated *ASL* in tumors (*p* = 0.005). 17% of patients had downregulated *ASS1* and 38% had it upregulated (*p* = 0.076). *ORNT1* was upregulated in 11% of patients as opposed to 59% with downregulated enzyme expression in tumors (*p* < 0.001). Tumor *DDAH1* was upregulated in 11% of patients and downregulated in 33% (*p* = 0.050) and *PRMT2* was upregulated in 15% of patients and downregulated in 33% (*p* = 0.120). Tumor *NOS2* expression was upregulated in 67% of patients and downregulated in 18.5% of patients (*p* < 0.001).

#### 3.2.2. Effect of Cancer Anatomical Site and Pathology on Pathway Enzymes

**Figure 3:**
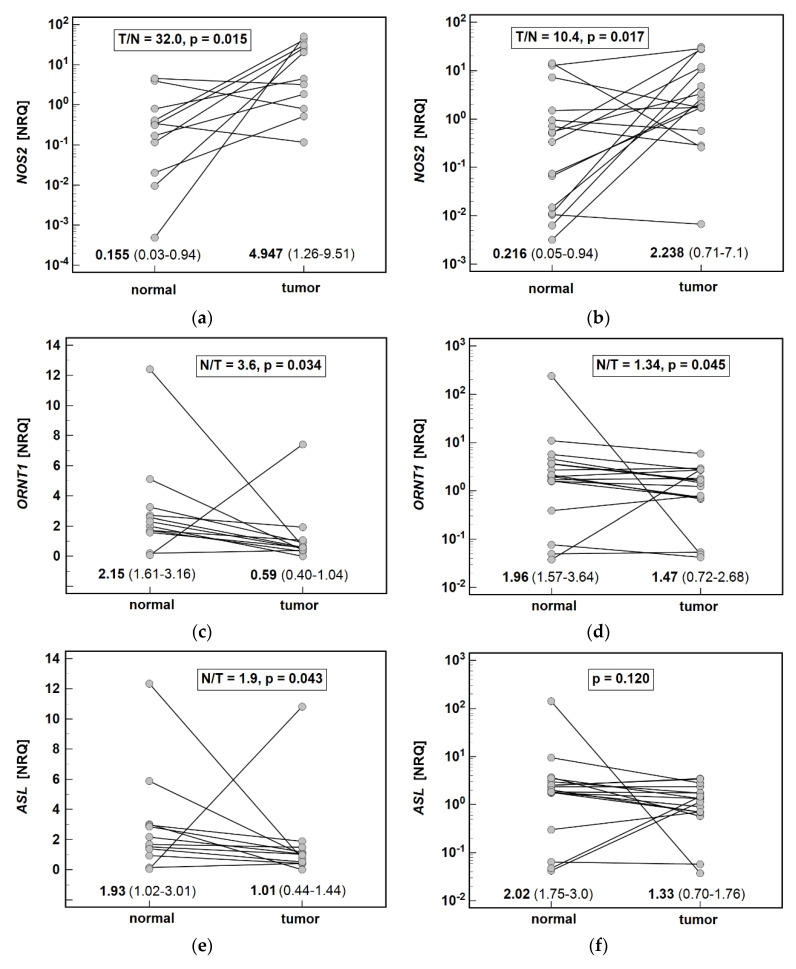

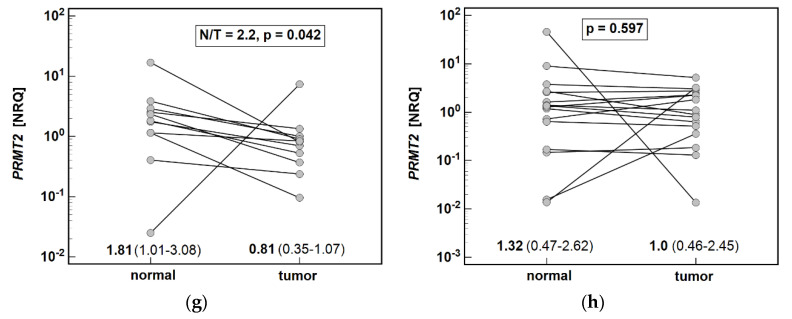
Effect of anatomical site on enzyme expression: (**a**) *NOS2* in cardia subtype of gastric adenocarcinoma (CA; *n* = 11); (**b**) *NOS2* in non-cardia subtype of gastric adenocarcinoma (GA; *n* = 16); (**c**) *ORNT1* in CA (*n* = 12); (**d**) *ORNT1* in GA (*n* = 17); (**e**) *ASL* in CA (*n* = 12); (**f**) *ASL* in GA (*n* = 17); (**g**) *PRMT2* in CA (*n* = 11); (**h**) *PRMT2* in GA (*n* = 11). Data analyzed using t-test for paired samples (*NOS2*) or Wilcoxon test and presented as geometric means or medians with 95% confidence interval. T/N, tumor-to-normal expression ratio; N/T, normal-to-tumor expression ratio; *NOS2*, inducible nitric oxide synthase; *ORNT1*, ornithine translocase 1; *ASL*, argininosuccinate lyase; *PRMT2*, protein arginine methyltransferase; NRQ, normalized relative quantities.

The upregulation of *NOS2* and the downregulation of *ORNT1* expression in tumors was markedly higher in CA than in GA. In turn, the downregulation of *ASL* and *PRMT2* in tumors was significant solely in CA (Figure 3).

**Figure 4:**
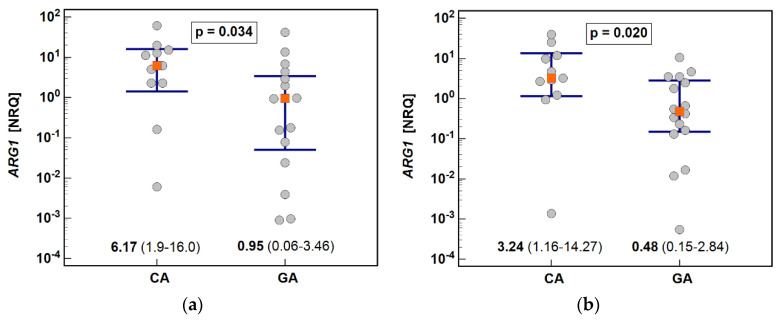
Effect of anatomical site on *ARG1* expression in: (**a**) macroscopically normal tumor-adjacent tissues; (**b**) tumors. Data presented as geometric means with 95% confidence interval and analyzed using t-test for independent samples. *ARG1*, arginase-1; NRQ, normalized relative quantities; CA, cardia subtype of gastric adenocarcinoma; GA, non-cardia subtype of gastric adenocarcinoma.

Anatomical site affected the expression of *ARG1*, in both normal and tumor tissue, which was higher by six-fold in CA than in GA (Figure 4).

Tumor expression of *NOS2* was significantly inversely correlated (ρ = −0.47, *p* = 0.014) with TNM stage (I-II-III-IV).

Tumor expression of *NOS2* was insignificantly (ρ = −0.35, *p* = 0.072) and these of *PRMT2* (ρ = −0.41, *p* = 0.036) and *ASL* (ρ = −0.41, *p* = 0.029) were significantly inversely correlated with depth of tumor invasion (T1/2–T3–T4).

**Figure 5:**
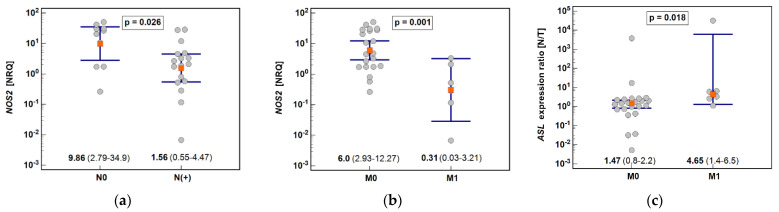
Effect of metastasis on *NOS2* and *ASL* expression: (**a**) Lymph node metastasis and tumor *NOS2* expression; (**b**) Distant metastasis and tumor *NOS2* expression; (**c**) Distant metastasis and *ASL* expression ratio. Data presented as geometric means and analyzed using t-test for independent samples (*NOS2*) or medians and analyzed using Mann–Whitney U test (*ASL*). *NOS2*, inducible nitric oxide synthase; *ASL*, argininosuccinate lyase; NRQ, normalized relative quantities; N/T, normal-to-tumor expression ratio.

Tumor expression of *NOS2* was decreased in patients with lymph node (by 6.3-fold) and distant metastasis (by 19.7-fold) compared to those without metastases (Figure 5).

In addition, *ASL* downregulation in tumors compared to normal tissue (N/T expression ratio) was more pronounced in M1 than M0 cancers (by 3.2-fold) (Figure 5).

#### 3.2.3. Interplay between Pathway Enzymes

The expression of *NOS2* was not significantly correlated with any other pathway gene in normal mucosa (Table 7), while its association with expression of other genes in tumors was moderate (Table 8). *ARG1* was rather poorly related with other pathway enzymes as well. The strongest correlations were between *PRMTs* and *DDAHs*, both in tumors and normal tissue, although those in normal tissue were generally stronger. The most marked cancer-related difference seems to be associated with *ODC* and *ARG2* expression patterns, mostly changed from very strong to strong (with *DDAHs*) or moderate (with *PRMTs*), and with loosening of *ASS1* and *ASL* association with each other as well as other pathway enzymes.

#### 3.2.4. Co-Expression with Markers of Proliferation, Survival, Inflammation, Angiogenesis, Metabolic Reprogramming, and Epithelial-Mesenchymal-Transition

The expression of pathway enzymes was—to varying degrees—interrelated with the expression of key genes involved in cancer growth and progression. In univariate analysis (Table 9), all genes except for *ASL* and *ORNT1* were positively correlated with proliferation index *Ki67* and anti-apoptotic *BCLXL*. *Ki67* was an independent predictor of *ARG2* and *PRMT1* expression, while *BCLXL* was a predictor of *DDAH1* and *PRMT5* in multiple regression. *CDKN1A*, encoding cell cycle regulator p21, was positively correlated with *ARG2*, *ODC1*, and *PRMT5* and was an independent predictor of *ODC*1 in multiple regression. Except for *ASS1*, the expression of all pathway enzymes was correlated with mediators of inflammation: *CCL2* or *PTGS2* (encoding COX2) or both, with *PTGS2* being an independent predictor of *ORNT1* and *CCL2* of *PRMT2* and *PRMT5*. As a marker of metabolic reprogramming, *GLUT1* was positively correlated with *ASS1*, of which it was an independent predictor in multiple regression, along with *ARG2*, *ODC1*, *PRMT1*, and *PRMT5*. Angiogenic *VEGFA* positively correlated with *ODC1*, *ORNT1*, *PRMT1*, and *PRMT5* and epithelial mesenchymal markers were related to the expression of *ARG2*, *DDAH1*, *DDAH2*, *ODC1*, and *PRMT*s. As *PRMT5* expression was independently associated with both *BCLXL* and *CCL2*, the respective partial correlation coefficients were: r_p_ = 0.79, *p* = 0.003 and r_p_ = 0.61, *p* = 0.035. *ARG1* and *NOS2* did not display any significant correlations.

## 4. Discussion

Metabolomic profiling is viewed as a promising approach to discover novel biomarkers facilitating cancer diagnosis and differentiation, monitoring treatment efficacy, and prognostication [5]. In the present study, the analysis of metabolites associated with arginine metabolism clearly indicated that amino acid deficiency occurs at systemic level already in patients with benign gastric disorders, and is further exacerbated in cardiac cancer. Moreover, depletion of citrulline and ornithine was aggravated in cancer patients with lymph node and distant metastases. Consistently, amino acid concentrations were negatively correlated with a set of immune modulators and proinflammatory cytokines as well as cancer-promoting growth factors. A gastric cancer-related systemic drop in arginine and citrulline, but not ornithine, has previously been shown by Miyagi et al. [21], and plasma-free amino acid profiling has been successfully explored as a diagnostic tool. Therefore, we assessed the individual discriminative power of arginine, ornithine, and citrulline and found ornithine to be superior cancer marker with overall accuracy exceeding 80%. Citrulline, in turn, was the only metabolite able to discriminate cancer patients by anatomical subsite of primary tumor, although its power was only moderate. Contrary to amino acids, the other evaluated metabolites—dimethylarginines and DMA—were elevated in patients with benign gastric disorders and SDMA and DMA also in cancer patients. Ornithine, ADMA, and SDMA were moderately efficient in discriminating patients with cancer from those with benign gastric disorders. Among assessed metabolites, DMA was the best individual marker of cancer presence. Still, we demonstrated that concomitant quantification of all metabolites was superior to individual determinations in terms of diagnostic power in overall cancer detection, differentiation between benign and cancerous gastric diseases, and distinguishing CA from GA with, respectively, 91%, 70%, and 66% overall accuracy.

Systemic arginine depletion was accompanied by upregulated local *NOS2* expression while the expression levels of *ARG*s were unaltered between tumors and adjacent mucosa. This observation is consistent with that of Wang et al. [22] who showed upregulated NOS2 in gastric tumors. However, it does not confirm previous findings on the upregulation of ARG in breast [23] and that of ODC in gastric [24] tumors. As NOS2 overexpression has been associated with transformed epithelial cells [22] and that of ARG and ODC with tumor-infiltrating macrophages [23,25], possible low content of these immune cells in tumors analyzed in the present study might account for lack of ARG and ODC upregulation. Unlike immunochemistry, the RTqPCR technique is fully quantitative but does not allow for determining the cellular source of expression. Noteworthily, lack of gene upregulation in tumors compared to adjacent tissue may not indicate lack of cancer-related gene upregulation. It has been repeatedly demonstrated that tumor-adjacent tissue might already have upregulated gene expression, even indicating comparative gene downregulation in tumors, despite lack of morphological and histological changes in its architecture [26,27,28,29]. Such apparent downregulation, resulting from less pronounced upregulation in tumors, has also been noted for *ARG1* expression in the colon [7]. No normal gastric mucosa was available in the current study to confirm the speculation, but the notion is supported by higher ODC activity in non-transformed mucosa from GC patients than normal mucosa from healthy individuals reported by others [24].

We confirmed, on a larger set of samples, our previous observation [19] that *NOS2* upregulation is greater in cardia subtype of gastric cancer. The downregulation of *ORNT1*, *ASL*, and *PRMT2* was more evident in cardia subtype as well. Considering the tumor-supporting consequences of enzyme deregulation, this finding might shed some light on molecular background of more aggressive phenotype and worse prognosis of cardia than non-cardia gastric cancer [30].

Elevated concentration of dimethylarginines, more so in benign disorders than in cancer, is in line with inflammatory character of SDMA and the role attributed to ADMA in gastric injury. ADMA has been shown to induce inflammatory response and oxidative stress in gastric mucosa [31,32] and mediate cell migration and invasion via Wnt/β-catenin signaling pathway [33]. The impact of SDMA accumulation is mostly unknown, but in colorectal tumors, it has been linked with greater metastatic potential [34]. Here, both ADMA and SDMA were positively correlated with systemic hepatocyte growth factor (HGF), pivotal for gastric cancer development and progression [35]. SDMA was also correlated with stroma-derived (SCF), the signaling of which is involved in viability and self-renewing properties of cancer stem cells [36]. The interrelationship between intermediates of arginine metabolic pathways and stemness-promoting cytokines, recurring in various cancers [9], is intriguing and worth exploration.

Corroborating findings of others [32,37], systemic elevation of ADMA was accompanied by *DDAH1* downregulation. A proneoplastic role has generally been attributed to DDAHs [12] and the only evidence of DDAH1 acting as a tumor suppressor has been shown in gastric cancer [12,37]. Still, a co-expression pattern of *DDAH1* observed here might imply a tumor-promoting role. *DDAH1* expression was positively correlated with genes encoding proliferation marker Ki67, anti-apoptotic BCLXL, and mesenchymal marker claudin-2 (*CLDN2*), with *BCLXL* being an independent predictor of *DDAH1* expression. Similar correlation patterns were observed for *DDAH2*, a dominant endothelial isoform.

DDAH activity yields citrulline and DMA. The status and role of DMA in gastric cancer is largely unknown. Nonetheless, its accumulation is likely disadvantageous as DMA is a precursor of nitrosodimethylamine—a suspected carcinogen—and can be effectively transported from blood into gastric fluid [38]. DMA in our patients was positively correlated with SDMA and with SCGFβ, a recently discovered secreted sulfated glycoprotein of unknown status and role in gastric cancer, which, however, is a marker of drug-resistance in lung and liver cancers [39].

ASS1 and ASL are involved in intracellular de novo synthesis of arginine from citrulline. Counterintuitively, however, number of cancers downregulate ASS1 and become arginine auxotrophic. The proposed advantage of ASS1 downregulation for tumors is the promotion of proliferation under normal conditions, greater invasiveness under hypoxia, and a buildup of glutamine under acidic conditions [40,41]. Herein, *ASS1* expression was not significantly affected but, in line with its proposed pro-survival role in gastric cancer, it was positively correlated with *Ki67* and *BCLXL*. Less is known about ASL, which is the only enzyme able to synthesize arginine endogenously and its silencing also results in arginine auxotrophy [15]. To the best of our knowledge, *ASL* expression in gastric cancer has not been investigated. Here, we found it to be downregulated in tumors by two-fold on average. Moreover, *ASL* downregulation was more pronounced in cancers metastasizing to distant organs and *ASL* expression in tumor was inversely correlated with depth of invasion. Downregulation of *ASL* is of clinical relevance as, if confirmed on a larger set of samples, it implies that gastric cancer, particularly the one located in cardia, might still be sensitive to arginine-deprivation therapies, despite *ASS1* overexpression.

Among cancer-related metabolic aberrations, dysregulation of urea cycle enzymes is quite prevalent and linked with worse overall prognosis but better response to immunotherapy based on checkpoint inhibitors [42]. As the enzymes of the cycle compete for nitrogenous substrates with others, loss-of-function mutations in genes encoding *ASS1* or *ASL* or ornithine translocase (*ORNT1*) facilitate pyrimidine synthesis by dihydrooratase, and results, as a consequence, in increased cell proliferation [42]. Therefore, downregulation of *ASL* combined with even more accentuated lower expression of *ORNT1* might potentially translate into metabolic rewiring promoting pyrimidine synthesis. In line with Lee et al.’s [42] observations, determining expression level of *ASL* and *ORNT1* might therefore help identify gastric cancer patients more likely to benefit from immune checkpoint inhibitor therapy.

Patients with benign gastric disorders had significantly higher systemic concentrations of dimethylarginines and SDMA was higher also in cancer patients. ADMA and SDMA are products of, respectively, type I and type II PRMTs, but dysregulation of dimethylarginines was not reflected locally and *PRMT1* (prototypical type I enzyme) and *PRMT5* (prototypical type II enzyme) expression levels were unaltered. PRMT upregulation in certain cancer types [9] has been noted and evoked an interest in PRMTs and their inhibitors as potential antineoplastic strategy [43,44]. Still, unaltered PRMT expression agrees well with the housekeeping nature of those enzymes, further underscored by their tight interrelationship observed here. It is worth mentioning, however, that lack of *PRMT1* and *PRMT5* elevation in tumors might not necessarily mean that enzyme expression is not affected by cancer. As we have previously shown in colorectal cancer, *PRMT1* and *PRMT5* expression can be upregulated both in colonic tumors and adjacent tissue [7].

Unlike main isoforms, tumor PRMT2 expression was clearly downregulated in CA and reflected the depth of tumor invasion. Contrary to PRMT1 and PRMT5, little is known about PRMT2 and its potential substrates. In fact, enzyme has even been suspected of lack of methyltransferase activity [45]. Nonetheless, it has been shown that PRMT2 may act as a coactivator for various receptors. However, its partners are implicated in opposing activities, either facilitating or inhibiting tumor growth [45]. While PRMT2 status and role in gastric cancer does not seem to be previously investigated, our observation on diminished PRMT2 expression in cardia tumors is in line with antitumor activity played by peroxisome proliferator-activated receptor γ (PPARγ) in gastric carcinogenesis [46], even though the receptor is reportedly upregulated in gastric tumors [47]. Moreover, it also agrees well with growth inhibition exerted by PRMT2 in breast cancer [48].

## 5. Conclusions

Metabolic reprogramming in gastric cancer is manifested by aberrant metabolism of arginine, reflecting cancer subtype and pathology, as well as by altered interplay of pathway intermediates and enzymes. Quantifying metabolites associated with arginine metabolism for diagnostic purposes holds promise, but requires validation prior to clinical application. Exploiting upregulation of *NOS2* and downregulation of *ASL*, *PRMT2*, and *ORNT*1 for therapeutic purposes requires confirmation on a larger set of samples. Previously not investigated status of *ASL*, *PRMT2*, and *ORNT*1 warrants further functional studies on the role and clinical significance of enzyme downregulation in gastric cancer.

## Figures and Tables

**Table 1:** Characteristics of study population for metabolomic analysis.

Characteristics:	Controls	Benign Disorders	Cardia Subtype	Non-Cardia Subtype	*p*
N	153	50	35	55	-
Sex (F/M), n	74/79	25/25	12/23	20/35	0.214 ^1^
Age (y), mean ± SD	59.8 ± 12	58.8 ± 14	59.3 ± 8	62.3 ± 12	0.455 ^2^
Stage (I/II/III/IV)	na	na	0/5/4/26	4/8/7/36	0.421 ^3^
Primary tumor, T (1/2/3/4)	na	na	0/2/7/26	5/3/14/33	0.249 ^3^
Lymph node metastasis, N (no/yes)	na	na	6/29	12/43	0.788 ^1^
Distant metastasis, M (no/yes)	na	na	9/26	19/36	0.485 ^1^

N, number of observations; F/M, female-to-male ratio; y, years; SD, standard deviation; *p*, probability value, with *p* < 0.05 indicative of statistical significance); ^1^ Fisher’s exact test; ^2^ one-way analysis of variance; ^3^ Chi-squared test; na, non-applicable.

**Table 2:** Characteristics of study population for analysis for transcriptomic analysis.

Characteristics:	All	GA	CA	*p*
N	29	17	12	-
Sex (F/M), n	16/13	10/7	6/6	0.716 ^1^
Age (y), mean (95% CI)	65.2 (62–69)	65.1 (60–70)	65.4 (61–70)	0.921 ^2^
Stage (I/II/III/IV)	4/6/13/6	3/3/7/4	1/3/6/2	0.823 ^3^
Primary tumor, T (1-2/3/4)	5/18/6	4/10/3	1/8/3	0.550 ^3^
Lymph node metastasis, N (no/yes)	11/18	6/11	5/7	1.0 ^1^
Distant metastasis, M (no/yes)	23/6	13/4	10/2	1.0 ^1^
Histological grade, G (1/2/3)	3/13/13	2/6/9	1/7/4	0.469 ^3^

N, number of observations; F/M, female-to-male ratio; y, years; CI, confidence interval; *p*, probability value, with *p* < 0.05 indicative of statistical significance); ^1^ Fisher’s exact test; ^2^ *t*-test for independent samples; ^3^ Chi-squared test; GA, non-cardia subtype of gastric adenocarcinoma; CA, cardia subtype of gastric adenocarcinoma.

**Table 3:** Effect of cancer pathology on metabolites associated with arginine metabolism.

Metabolite	TNM	T	N0 vs. N(+)	M0 vs. M1
Cit	ns	ns	31.0 ± 13 vs. 25.6 ± 9.4, *p* = 0.048 ^2^	30.5 ± 11 vs. 25.0 ± 9.7, *p* = 0.018 ^2^
Orn	ρ = −0.26, *p* = 0.012 ^1^	ρ = −0.22, *p* = 0.040 ^1^	89.0 ± 23.1 vs. 70.2 ± 22.6, *p* = 0.002 ^2^	ns
ADMA	ρ = −0.21, *p* = 0.047 ^1^	ρ = −0.25, *p* = 0.017 ^1^	ns	ns

Data analyzed using: ^1^ Spearman rank correlation and presented as correlation coefficient rho (ρ); ^2^ *t*-test for independent samples and presented as means ± standard deviation. TNM, tumor-node-metastasis cancer staging system (I = 4/II = 13/III = 11/IV = 62); T, depth of tumor invasion (T1 = 5/T2 = 5/T3 = 21/T4 = 59); N, lymph node metastasis (N0 = 18/N(+) = 72); M, distant metastasis (M0 = 28/M1 = 62); Cit, citrulline; Orn, ornithine; ADMA, asymmetric dimethylarginine; ns, non-significant (*p* > 0.05).

**Table 4:** Correlation patterns in cancer patients between metabolites associated with arginine metabolism and mediators of inflammatory and immune responses and angiogenesis.

Metabolite	Cytokine	*n*	ρ	Cytokine	*n*	ρ
Arg	IL-2	28	−0.49 ^2^	EOX1	82	0.24 ^1^
	IL-15	23	−0.51 ^1^	MIF	26	−0.57 ^2^
	IL-16	26	−0.42 ^1^	SDF1α	26	−0.55 ^2^
Cit	IL-4	82	−0.26 ^1^	FGF2	82	−0.36 ^3^
	IL-6	82	−0.33 ^2^	GM-CSF	82	−0.23 ^1^
	IL-7	82	−0.23 ^1^	PDGF-BB	82	−0.23 ^1^
	IL-10	82	−0.26 ^1^	VEGF-A	82	−0.26 ^1^
	IL-18	26	−0.53 ^2^	TRAIL	26	−0.43 ^1^
Orn	IL-1β	82	−0.34 ^2^	IL-17	82	−0.41 ^3^
	IL-4	82	−0.25 ^1^	G-CSF	82	−0.25 ^1^
	IL-6	82	−0.24 ^1^	IFNγ	82	−0.30 ^2^
	IL-7	82	−0.38 ^3^	MIP-1α	82	−0.33 ^2^
	IL-10	82	0.22 ^1^	PDGF-BB	82	−0.26 ^1^
	IL-16	26	0.40 ^1^			
ADMA	G-CSF	82	0.26 ^1^	IP-10	82	0.22 ^1^
	HGF	26	0.41 ^1^	MIP-1α	82	0.23 ^1^
	IFNγ	82	0.29 ^2^			
SDMA	HGF	26	0.42 ^1^	SCF	26	0.40 ^1^
DMA	IP-10	82	0.29 ^2^	RANTES	82	0.23 ^1^
	MIF	26	−0.43 ^1^	SCGFβ	26	0.64 ^3^
	MIP-1β	82	0.22 ^1^			

Data presented as Spearman correlation coefficients rho (ρ). Statistical significance is indicated as follows: ^1^ *p* < 0.05; ^2^ *p* ≤ 0.01; ^3^ *p* ≤ 0.001. *N*, number of observations; Arg, arginine; Cit, citrulline; Orn, ornithine; ADMA, asymmetric dimethylarginine; SDMA, symmetric dimethylarginine; DMA, dimethylamine; IL, interleukin; EOX1, eotaxin-1; MIF, macrophage migration inhibitory factor; SDF1α, stromal cell-derived factor-1α; FGF2, fibroblast growth factor-2; GM-CSF, granulocyte-macrophage colony-stimulating factor; PDGF-BB, platelet-derived growth factor-BB; VEGF-A, vascular endothelial growth factor-A; TRAIL, tumor necrosis factor-related apoptosis inducing ligand; G-CSF, granulocyte colony-stimulating factor; IFN, interferon; MIP-1, monocyte inflammatory protein-1; HGF, hepatocyte growth factor; IP-10, IFNγ-induced protein 10; SCF, stem cell factor; RANTES, regulated upon activation, normal T-cell expressed, and secreted (CCL5); SCGFβ, stem cell growth factor-β.

**Table 5:** Diagnostic power of metabolites associated with arginine metabolism as gastric cancer biomarkers.

Metabolite	Parameter	Cancers vs. CTRL	Cancer vs. BN	CA vs. GA
Arg	AUC (95%CI)	0.770 (0.71–0.82) ^1^	ns	ns
	criterion	≤142.9 μM		
	sens. and spec.	88.9 and 58.2		
Cit	AUC (95%CI)	0.758 (0.70–0.81) ^1^	ns	0.631 (0.52–0.73) ^3^
	criterion	≤37.7 μM		≤29.2 μM
	sens. and spec.	91.1 and 55.6		82.9 and 41.8
Orn	AUC (95%CI)	0.821 (0.74–0.89) ^1^	0.648 (0.56–0.73) ^2^	ns
	criterion	≤91.8 μM	≤70.6 μM	
	sens. and spec.	84.4 and 72.0	54.4 and 78.0	
ADMA	AUC (95%CI)	ns	0.654 (0.57–0.73) ^2^	ns
	criterion		≤0.454 μM	
	sens. and spec.		70.0 and 62.0	
SDMA	AUC (95%CI)	0.638 (0.57–0.70) ^1^	0.629 (0.54–0.71) ^2^	ns
	criterion	>0.356 μM	≤0.373 μM	
	sens. and spec.	55.6 and 72.5	53.3 and 72.0	
DMA	AUC (95%CI)	0.887 (0.84–0.92) ^1^	ns	ns
	criterion	>1.5 μM		
	sens. and spec.	93.3 and 73.2		
Panel (all metabolites)	AUC (95%CI)	0.914 (0.85–0.96) ^1^	0.697 (0.61–0.77) ^1^	0.659 (0.55–0.76) ^2^
	criterion	>0.659 ^4^	>0.634 ^4^	>0.366 ^4^
	sens. and spec.	91.1 and 88.0	76.6 and 68.0	77.1 and 66.0

Data were analyzed using receiver operating characteristics (ROC) curve analysis. Data presented as area under ROC curve (AUC), indicative of marker overall accuracy, with 95% confidence interval and statistical significance denoted as: ^1^ *p* < 0.001; ^2^ *p* < 0.01; ^3^ *p* < 0.05. ^4^ predicted probabilities calculated in logistic regression analysis. CTRL, controls; BN, patients with benign gastric disorders; CA, cardiac adenocarcinoma; GC, gastric adenocarcinoma; sens., sensitivity; spec., specificity; ns, non-significant; Arg, arginine; Cit, citrulline; Orn, ornithine; ADMA, asymmetric dimethylarginine; SDMA, symmetric dimethylarginine; DMA, dimethylamine.

**Table 6:** Expression of genes associated with arginine metabolic pathways in gastric normal mucosa and patient-matched tumors.

Gene	Normal Mean (95%*CI*) [NRQ]	Tumor Mean (95%*CI*) [NRQ]	*p* Value	Expression Ratio T/N (N/T)
*ASL*	2.02 (1.65–2.88)	1.05 (0.70–1.48)	0.010 ^1^	0.52 (1.9)
*ARG1*	2.29 (0.18–6.20)	1.77 (0.42–3.47)	0.456 ^1^	-
*ARG2*	1.08 (0.76–1.62)	0.86 (0.55–2.35)	0.981 ^1^	-
*ASS1*	1.16 (0.72–1.36)	1.62 (0.84–1.87)	0.071 ^1^	-
*DDAH1*	1.12 (1.01–1.69)	0.70 (0.55–0.96)	0.026 ^1^	0.62 (1.6)
*DDAH2*	1.27 (0.84–1.84)	1.10 (0.50–1.77)	0.737 ^1^	-
*NOS2*	0.19 (0.07–0.54)	3.09 (1.34–7.13)	<0.001 ^2^	16.4
*ODC1*	1.24 (0.67–1.71)	0.81 (0.63–1.4)	0.400 ^1^	-
*ORNT1*	2.00 (1.67–2.84)	0.79 (0.59–1.62)	0.003 ^1^	0.39 (2.5)
*PRMT1*	1.49 (0.96–1.96)	1.06 (0.71–1.50)	0.524 ^1^	-
*PRMT2*	1.39 (1.13–2.53)	0.84 (0.53–1.37)	0.037 ^1^	0.60 (1.7)
*PRMT5*	1.19 (0.90–1.72)	0.92 (0.68–1.26)	0.249 ^1^	-

Data presented as medians or geometric means (*NOS2*) with 95% confidence interval (CI). *ASL*, argininosuccinate lyase; *ARG*, arginase; *ASS1*, argininosuccinate synthase 1; *DDAH*, dimethylarginine dimethylaminohydrolase; *NOS*, nitric oxide synthase; *ODC*, ornithine decarboxylase; *ORNT1*, ornithine translocase 1; *PRMT*, protein arginine methyltransferase. ^1^ Wilcoxon test; ^2^ t-test for paired samples; T/N, tumor-to-normal expression ratio; N/T, normal-to-tumor expression ratio; NRQ, normalized relative quantities, calculated as individual sample Cq values subtracted from geometric mean of all Cq values (ΔCq), linearized by 2^^ΔCq^ conversion and normalized to the expression of reference gene [18].

**Table 7:** Correlation patterns between enzymes associated with arginine metabolism in normal mucosa from cancer patients.

Gene	*ODC1*	*DDAH1*	*ARG2*	*PRMT1*	*DDAH2*	*ORNT1*	*PRMT2*	*ASL*	*ASS1*	*ARG1*	*NOS2*
*PRMT5*	0.89 ^1^	0.88 ^1^	0.90 ^1^	0.93 ^1^	0.86 ^1^	0.77 ^1^	0.81 ^1^	0.75 ^1^	0.74 ^1^	0.54 ^3^	ns
*ODC1*		0.80 ^1^	0.93 ^1^	0.78 ^1^	0.77 ^1^	0.83 ^1^	0.71 ^1^	0.74 ^1^	0.73 ^1^	0.53 ^3^	ns
*DDAH1*			0.85 ^1^	0.85 ^1^	0.71 ^1^	0.78 ^1^	0.73 ^1^	0.82 ^1^	0.75 ^1^	ns	ns
*ARG2*				0.82 ^1^	0.73 ^1^	0.74 ^1^	0.71 ^1^	0.67 ^1^	0.65 ^2^	0.56 ^3^	ns
*PRMT1*					0.86 ^1^	0.70 ^1^	0.81 ^1^	0.66 ^1^	0.62 ^2^	0.44 ^4^	ns
*DDAH2*						0.70 ^1^	0.93 ^1^	0.64 ^2^	0.67 ^2^	0.51 ^3^	ns
*ORNT1*							0.73 ^1^	0.86 ^1^	0.81 ^1^	0.45 ^4^	ns
*PRMT2*								0.72 ^1^	0.71 ^1^	0.39 ^4^	ns
*ASL*									0.82 ^1^	ns	ns
*ASS1*										0.45 ^4^	ns
*ARG1*											ns

Data presented as a heatmap of Spearman correlation coefficients (ρ) with statistical significance indicated as follows: ^1^ *p* ≤ 0.0001; ^2^ *p* ≤ 0.001; ^3^ *p* ≤ 0.01; ^4^ *p* < 0.05 and strength of association color-coded ranging from red (strong positive), to yellow, green, and blue (strong negative). *ASL*, argininosuccinate lyase; *ARG*, arginase; *ASS1*, argininosuccinate synthase 1; *DDAH*, dimethylarginine dimethylaminohydrolase; *NOS*, nitric oxide synthase; *ODC*, ornithine decarboxylase; *ORNT1*, ornithine translocase 1; *PRMT*, protein arginine methyltransferase; ns, not significant (*p* ≥ 0.05).

**Table 8:** Correlation patterns between enzymes associated with arginine metabolism in cardiac and gastric tumors.

	*PRMT5*	*PRMT1*	*DDAH1*	*PRMT2*	*ORNT1*	*ODC1*	*ARG2*	*ASL*	*ASS1*	*NOS2*	*ARG1*
*DDAH2*	0.79 ^1^	0.79 ^1^	0.66 ^2^	0.75 ^1^	0.63 ^2^	0.70 ^1^	0.74 ^1^	0.56 ^3^	0.51 ^3^	0.51 ^3^	0.43 ^4^
*PRMT5*		0.93 ^1^	0.81 ^1^	0.87 ^1^	0.66 ^2^	0.52 ^3^	0.57 ^3^	0.60 ^2^	0.43 ^4^	ns	ns
*PRMT1*			0.79 ^1^	0.78 ^1^	0.58 ^2^	0.52 ^3^	0.62 ^2^	0.49 ^3^	0.49 ^3^	0.42 ^4^	ns
*DDAH1*				0.64 ^2^	0.66 ^2^	0.60 ^2^	0.54 ^3^	0.65 ^2^	0.39 ^4^	ns	ns
*PRMT2*					0.51 ^3^	0.39 ^4^	0.49 ^3^	0.63 ^2^	ns	ns	ns
*ORNT1*						0.67 ^1^	0.48 ^4^	0.75 ^1^	0.37 ^4^	0.51 ^3^	ns
*ODC1*							0.77 ^1^	0.48 ^4^	0.52 ^3^	0.43 ^4^	0.46 ^4^
*ARG2*								ns	0.55 ^3^	ns	0.45 ^4^
*ASL*									ns	0.55 ^3^	ns
*ASS1*										ns	0.66 ^2^
*NOS2*											0.39 ^4^

Data presented as a heatmap of Spearman correlation coefficients (ρ) with statistical significance indicated as follows: ^1^ *p* ≤ 0.0001; ^2^ *p* ≤ 0.001; ^3^ *p* ≤ 0.01; ^4^ *p* < 0.05 and strength of association color-coded ranging from red (strong positive) to yellow, green, and blue (strong negative). *ASL*, argininosuccinate lyase; *ARG*, arginase; *ASS1*, argininosuccinate synthase 1; *DDAH*, dimethylarginine dimethylaminohydrolase; *NOS*, nitric oxide synthase; *ODC*, ornithine decarboxylase; *ORNT1*, ornithine translocase 1; *PRMT*, protein arginine methyltransferase; ns, not significant (*p* ≥ 0.05).

**Table 9:** Correlation patterns between genes encoding pathway enzymes and key cancer-related molecules.

Gene	*ASS1*	*ARG2*	*ASL*	*DDAH1*	*DDAH2*	*ODC1*	*ORNT1*	*PRMT1*	*PRMT2*	*PRMT5*
*Ki67*	0.68 ^2^	0.72 ^2^	ns	0.70 ^2^	0.71 ^2^	0.61 ^3^	ns	0.72 ^2^	0.73 ^2^	0.85 ^1^
*HIF1A*	0.56 ^3^	0.70 ^2^	ns	ns	0.76 ^2^	ns	ns	0.60 ^3^	0.77 ^2^	0.84 ^1^
*CDKN1A*	ns	0.60 ^3^	ns	ns	ns	0.67 ^3^	ns	ns	ns	0.67 ^3^
*BCLXL*	0.54 ^3^	0.69 ^2^	ns	0.74 ^2^	0.75 ^2^	0.58 ^3^	ns	0.72 ^2^	0.73 ^2^	0.88 ^1^
*PTGS2*	ns	ns	0.57 ^3^	ns	ns	0.61 ^3^	0.65 ^2^	0.60 ^3^	0.59 ^3^	0.73 ^2^
*CCL2*	ns	0.61 ^3^	ns	0.56 ^3^	0.76 ^2^	ns	ns	ns	0.81 ^1^	0.80 ^1^
*GLUT1*	0.84 ^1^	0.64 ^3^	ns	ns	ns	0.56 ^3^	ns	0.64 ^2^	ns	0.66 ^3^
*VEGFA*	ns	ns	ns	ns	ns	0.64 ^3^	0.58 ^3^	0.67 ^2^	ns	0.70 ^2^
*CLDN2*	ns	ns	ns	0.65 ^3^	0.57 ^3^	ns	ns	0.58 ^3^	0.59 ^3^	0.75 ^2^
*TJP1*	ns	0.65 ^3^	ns	ns	0.63 ^3^	0.56 ^3^	ns	ns	0.66 ^3^	0.63 ^3^

Data presented as Pearson correlation coefficients (r) with statistical significance indicated as follows: ^1^ *p* ≤ 0.001; ^2^ *p* ≤ 0.01; ^3^ *p* < 0.05. *ASL*, argininosuccinate lyase; *ARG*, arginase; *ASS1*, argininosuccinate synthase 1; *DDAH*, dimethylarginine dimethylaminohydrolase; *NOS*, nitric oxide synthase; *ODC*, ornithine decarboxylase; *ORNT1*, ornithine translocase 1; *PRMT*, protein arginine methyltransferase; *Ki67*, proliferation marker Ki67; *HIF1A*, hypoxia-inducible factor 1A; *CDKN1A*, p21^WAF1/CIP1^; *BCLXL*, B-cell lymphoma-extra large; *CCL2*, monocyte chemoattractant protein (MCP)-1; *PTGS2*, cyclooxygenase-2; *GLUT1*, glucose transporter 1; *VEGFA*, vascular endothelial growth factor A; *CLDN2*, claudin-2; *TJP1*, zonula occludens-1; ns, not significant (*p* ≥ 0.05). Genes returned as independent predictors in multivariate analysis (multiple regression, stepwise method) are underlined.

## Data Availability

Data sharing not applicable.

## References

[1] Sitarz R., Skierucha M., Mielko J., Offerhaus G.J.A., Maciejewski R., Polkowski W.P. (2018). Gastric cancer: Epidemiology, prevention, classification, and treatment. Cancer Manag. Res..

[2] Liu X., Meltzer S.J. (2017). Gastric Cancer in the Era of Precision Medicine. Cell. Mol. Gastroenterol. Hepatol..

[3] Hanahan D., Weinberg R.A. (2011). Hallmarks of Cancer: The Next Generation. Cell.

[4] Torresano L., Nuevo-Tapioles C., Santacatterina F., Cuezva J.M. (2020). Metabolic reprogramming and disease progression in cancer patients. Biochim. Biophys. Acta Mol. Basis Dis..

[5] Xiao S., Zhou L. (2017). Gastric cancer: Metabolic and metabolomics perspectives (Review). Int. J. Oncol..

[6] Faubert B., Solmonson A., DeBerardinis R.J. (2020). Metabolic reprogramming and cancer progression. Science.

[7] Krzystek-Korpacka M., Szczęśniak-Sięga B., Szczuka I., Fortuna P., Zawadzki M., Kubiak A., Mierzchała-Pasierb M., Fleszar M.G., Lewandowski Ł., Serek P. (2020). L-Arginine/Nitric Oxide Pathway Is Altered in Colorectal Cancer and Can Be Modulated by Novel Derivatives from Oxicam Class of Non-Steroidal Anti-Inflammatory Drugs. Cancers.

[8] Bednarz-Misa I., Fleszar M.G., Zawadzki M., Kapturkiewicz B., Kubiak A., Neubauer K., Witkiewicz W., Krzystek-Korpacka M. (2020). L-Arginine/NO Pathway Metabolites in Colorectal Cancer: Relevance as Disease Biomarkers and Predictors of Adverse Clinical Outcomes Following Surgery. J. Clin. Med..

[9] Bednarz-Misa I., Fortuna P., Fleszar M.G., Lewandowski Ł., Diakowska D., Rosińczuk J., Krzystek-Korpacka M. (2020). Esophageal Squamous Cell Carcinoma Is Accompanied by Local and Systemic Changes in L-arginine/NO Pathway. Int. J. Mol. Sci..

[10] Szefel J., Danielak A., Kruszewski W.J. (2019). Metabolic pathways of L-arginine and therapeutic consequences in tumors. Adv. Med. Sci..

[11] Clemente G.S., van Waarde A.F., Antunes I., Dömling A.H., Elsinga P. (2020). Arginase as a Potential Biomarker of Disease Progression: A Molecular Imaging Perspective. Int. J. Mol. Sci..

[12] Hulin J.A., Gubareva E.A., Jarzebska N., Rodionov R.N., Mangoni A.A., Tommasi S. (2020). Inhibition of Dimethylarginine Dimethylaminohydrolase (DDAH) Enzymes as an Emerging Therapeutic Strategy to Target Angiogenesis and Vasculogenic Mimicry in Cancer. Front. Oncol..

[13] Al-Koussa H., El Mais N., Maalouf H., Abi-Habib R., El-Sibai M. (2020). Arginine deprivation: A potential therapeutic for cancer cell metastasis? A review. Cancer Cell Int..

[14] Nanthakumaran S., Brown I., Heys S.D., Schofield A.C. (2009). Inhibition of gastric cancer cell growth by arginine: Molecular mechanisms of action. Clin. Nutr..

[15] Keshet R., Erez A. (2018). Arginine and the metabolic regulation of nitric oxide synthesis in cancer. Dis. Models Mech..

[16] Fulton M.D., Brown T., Zheng Y.G. (2019). The Biological Axis of Protein Arginine Methylation and Asymmetric Dimethylarginine. Int. J. Mol. Sci..

[17] Fleszar M.G., Wiśniewski J., Krzystek-Korpacka M., Misiak B., Frydecka D., Piechowicz J., Lorenc-Kukuła K., Gamian A. (2018). Quantitative Analysis of l-Arginine, Dimethylated Arginine Derivatives, l-Citrulline, and Dimethylamine in Human Serum Using Liquid Chromatography-Mass Spectrometric Method. Chromatographia.

[18] Hellemans J., Vandesompele J., Kennedy S., Oswald N. (2011). qPCR data analysis—Unlocking the secret to successful results. PCR Troubleshooting and Optimization: The Essential Guide.

[19] Bednarz-Misa I., Fortuna P., Diakowska D., Jamrozik N., Krzystek-Korpacka M. (2020). Distinct Local and Systemic Molecular Signatures in the Esophageal and Gastric Cancers: Possible Therapy Targets and Biomarkers for Gastric Cancer. Int. J. Mol. Sci..

[20] Schober P., Boer C., Schwarte L.A. (2018). Correlation Coefficients: Appropriate Use and Interpretation. Anesth. Analg..

[21] Miyagi Y., Higashiyama M., Gochi A., Akaike M., Ishikawa T., Miura T., Saruki N., Bando E., Kimura H., Imamura F. (2011). Plasma free amino acid profiling of five types of cancer patients and its application for early detection. PLoS ONE.

[22] Wang Y.Z., Cao Y.Q., Wu J.N., Chen M., Cha X.Y. (2005). Expression of nitric oxide synthase in human gastric carcinoma and its relation to p53, PCNA. World J. Gastroenterol..

[23] Kus K., Kij A., Zakrzewska A., Jasztal A., Stojak M., Walczak M., Chlopicki S. (2018). Alterations in arginine and energy metabolism, structural and signalling lipids in metastatic breast cancer in mice detected in plasma by targeted metabolomics and lipidomics. Breast Cancer Res..

[24] Okuzumi J., Yamane T., Kitao Y., Tokiwa K., Yamaguchi T., Fujita Y., Nishino H., Iwashima A., Takahashi T. (1991). Increased mucosal ornithine decarboxylase activity in human gastric cancer. Cancer Res..

[25] Chaturvedi R., de Sablet T., Coburn L.A., Gobert A.P., Wilson K.T. (2012). Arginine and polyamines in Helicobacter pylori-induced immune dysregulation and gastric carcinogenesis. Amino Acids.

[26] Mao L., Clark D. (2015). Molecular margin of surgical resections—Where do we go from here?. Cancer.

[27] Dakubo G.D., Jakupciak J.P., Birch-Machin M.A., Parr R.L. (2007). Clinical implications and utility of field cancerization. Cancer Cell Int..

[28] Neubauer K., Bednarz-Misa I., Diakowska D., Kapturkiewicz B., Gamian A., Krzystek-Korpacka M. (2015). Nampt/PBEF/visfatin upregulation in colorectal tumors, mirrored in normal tissue and whole blood of colorectal cancer patients, is associated with metastasis, hypoxia, IL1β, and anemia. BioMed Res. Int..

[29] Krzystek-Korpacka M., Gorska S., Diakowska D., Kapturkiewicz B., Podkowik M., Gamian A., Bednarz-Misa I. (2017). Midkine is up-regulated in both cancerous and inflamed bowel, reflecting lymph node metastasis in colorectal cancer and clinical activity of ulcerative colitis. Cytokine.

[30] Asplund J., Kauppila J.H., Mattsson F., Lagergren J. (2018). Survival Trends in Gastric Adenocarcinoma: A Population-Based Study in Sweden. Ann. Surg. Oncol..

[31] Kwiecien S., Ptak-Belowska A., Krzysiek-Maczka G., Targosz A., Jasnos K., Magierowski M., Szczyrk U., Brzozowski B., Konturek S.J., Konturek P.C. (2012). Asymmetric dimethylarginine, an endogenous inhibitor of nitric oxide synthase, interacts with gastric oxidative metabolism and enhances stress-induced gastric lesions. J. Physiol. Pharmacol..

[32] Wang L., Zhou Y., Peng J., Zhang Z., Jiang D.J., Li Y.J. (2008). Role of endogenous nitric oxide synthase inhibitor in gastric mucosal injury. Can. J. Physiol. Pharmacol..

[33] Guo Q., Xu J., Huang Z., Yao Q., Chen F., Liu H., Zhang Z., Lin J. (2021). ADMA mediates gastric cancer cell migration and invasion via Wnt/β-catenin signaling pathway. Clin. Transl. Oncol..

[34] Brankovic B., Stanojevic G., Stojanovic I., Veljkovic A., Kocic G., Janosevic P., Nestorovic M., Petrovic D., Djindjic B., Pavlovic D. (2017). Nitric oxide synthesis modulation—A possible diagnostic and therapeutic target in colorectal cancer. J. BUON.

[35] Anestis A., Zoi I., Karamouzis M.V. (2018). Current advances of targeting HGF/c-Met pathway in gastric cancer. Ann. Transl. Med..

[36] Mazzoldi E.L., Pavan S., Pilotto G., Leone K., Pagotto A., Frezzini S., Nicoletto M.O., Amadori A., Pastò A. (2019). A juxtacrine/paracrine loop between C-Kit and stem cell factor promotes cancer stem cell survival in epithelial ovarian cancer. Cell Death Dis..

[37] Ye J., Xu J., Li Y., Huang Q., Huang J., Wang J., Zhong W., Lin X., Chen W., Lin X. (2017). DDAH1 mediates gastric cancer cell invasion and metastasis via Wnt/β-catenin signaling pathway. Mol. Oncol..

[38] Zeisel S.H., DaCosta K.A., Edrise B.M., Fox J.G. (1986). Transport of dimethylamine, a precursor of nitrosodimethylamine, into stomach of ferret and dog. Carcinogenesis.

[39] Sukowati C.H.C., Patti R., Pascut D., Ladju R.B., Tarchi P., Zanotta N., Comar M., Tiribelli C., Crocè L.S. (2018). Serum Stem Cell Growth Factor Beta for the Prediction of Therapy Response in Hepatocellular Carcinoma. BioMed Res. Int..

[40] Rabinovich S., Adler L., Yizhak K., Sarver A., Silberman A., Agron S., Stettner N., Sun Q., Brandis A., Helbling D. (2015). Diversion of aspartate in ASS1-deficient tumours fosters de novo pyrimidine synthesis. Nature.

[41] Silberman A., Goldman O., Boukobza Assayag O., Jacob A., Rabinovich S., Adler L., Lee J.S., Keshet R., Sarver A., Frug J. (2019). Acid-Induced Downregulation of ASS1 Contributes to the Maintenance of Intracellular pH in Cancer. Cancer Res..

[42] Lee J.S., Adler L., Karathia H., Carmel N., Rabinovich S., Auslander N., Keshet R., Stettner N., Silberman A., Agemy L. (2018). Urea Cycle Dysregulation Generates Clinically Relevant Genomic and Biochemical Signatures. Cell.

[43] Yang Y., Bedford M.T. (2013). Protein arginine methyltransferases and cancer. Nat. Rev. Cancer.

[44] Li X., Wang C., Jiang H., Luo C. (2019). A patent review of arginine methyltransferase inhibitors (2010–2018). Expert Opin. Ther. Pat..

[45] Baldwin R.M., Morettin A., Côté J. (2014). Role of PRMTs in cancer: Could minor isoforms be leaving a mark?. World J. Biol. Chem..

[46] Sato H., Ishihara S., Kawashima K., Moriyama N., Suetsugu H., Kazumori H., Okuyama T., Rumi M.A., Fukuda R., Nagasue N. (2000). Expression of peroxisome proliferator-activated receptor (PPAR)gamma in gastric cancer and inhibitory effects of PPARgamma agonists. Br. J. Cancer.

[47] Pazienza V., Vinciguerra M., Mazzoccoli G. (2012). PPARs Signaling and Cancer in the Gastrointestinal System. PPAR Res..

[48] Zhong J., Chen Y.J., Chen L., Shen Y.Y., Zhang Q.H., Yang J., Cao R.X., Zu X.Y., Wen G.B. (2017). PRMT2β, a C-terminal splice variant of PRMT2, inhibits the growth of breast cancer cells. Oncol. Rep..

